# Synthetic Polymers as Drug-Delivery Vehicles in Medicine

**DOI:** 10.1155/2008/469531

**Published:** 2008-05-08

**Authors:** Eberhard W. Neuse

**Affiliations:** School of Chemistry, University of the Witwatersrand, Private Bag 3, WITS 2050, Johannesburg, South Africa

## Abstract

Cancerous diseases present a formidable health problem worldwide. While the chemotherapy of cancer, in conjunction with other treatment modalities, has reached a significant level of maturity, efficacious use of such agents is still restricted by numerous pharmacological deficiencies, such as poor water solubility, short serum circulation lifetimes, and low bioavailability resulting from lack of affinity to cancer tissue and inadequate mechanisms of cell entry. More critically still, most drugs suffer from toxic side effects and a risk of drug resistance. The class of platinum anticancer drugs, although outstandingly potent, is particularly notorious in that respect. Among the countless methods developed in recent years in an effort to overcome these deficiencies, the technology of polymer-drug conjugation stands out as a particularly advanced treatment modality. The strategy involves the bioreversible binding, conjugating, of a medicinal agent to a water-soluble macromolecular carrier. Following pharmacokinetic pathways distinctly different from those of the common, nonpolymeric drugs, the conjugate so obtained will act as a prodrug providing safe transport of the bioactive agent to and into the affected, that is, cancerous cell for its ultimate cell-killing activity. The present treatise will acquaint us with the pharmacological fundamentals of this drug delivery approach, applied here specifically to the metalorganic platinum-type drug systems and the organometallic ferrocene drug model. We will see just how this technology leads to conjugates distinctly superior in antiproliferative activity to cisplatin, a clinically used antitumor agent used here as a standard. Polymer-drug conjugation involving metal-based and other medicinal agents has unquestionably matured to a practical tool to the pharmaceutical scientist, and all indications point to an illustrious career for this nascent drug delivery approach in the fight against cancer and other human maladies.

## 1. INTRODUCTION

The delivery of medicinal agents to the human body for healing purposes is probably as old as mankind. While in the early days such agents, now generally known as *drugs*, were simply delivered from hand to mouth, perhaps also by rubbing into the skin, or placing on an open wound, we are using more numerous and doubtlessly more complicated modalities of delivery in modern times, simply prompted by the need to control and combat a hugely increased variety of identified infirmities. A new stage of chemotherapeutic development was attained, more than a quarter of a century ago, with the advent of the polymer-drug conjugation paradigm, which teaches the use of polymeric, *that is,* macromolecular compounds as pivotal helpers in drug delivery. Numerous delivery techniques, taking advantage of the functional assistance provided by a macromolecular partner, have since been successfully developed for various routes of administration, not only the time-proven oral route (“just swallow it!”), but more importantly the parenteral routes involving intravenous, intraperitoneal, or intramuscular methods. It is particularly these parenteral administration techniques that have led to the successful use of partaking polymers. We will learn more about those developments further down the road in this paper. Although metal-free drug systems will find due mentioning in this treatise, emphasis will be on metal-containing compounds in accordance with this journal's mission. Before we delve into this core topic, we are prudently advised to take a primer lesson on the pharmacokinetic pathways generally available to a drug—any drug—after its administration to the mammalian organism, especially the human body. Section 2 will guide us leisurely on this learning path.

## 2. WHAT WE SHOULD KNOW ABOUT DRUG ADMINISTRATION

### 2.1. The cancer problem

Malignancies and cardiac diseases together provide the main cause of death in the developed world. In the United States, one in every three persons currently alive is estimated to contract some form of cancer, and every fifth person will ultimately succumb to it. A similar pattern of mortality obtains in other Western nations, and an even gloomier prognosis is observed in many developing countries. In South Africa, for example, cancer is the second-most common cause of death in the white, colored, and Asian population, and the third-most common cause in the black population group. Despite major variations among the different racial groups and incomplete registries especially in the black group, statistical data leave no doubt that, on average, South African rates for certain malignancies such as cervical, oesophageal, and skin cancers rank among the highest worldwide. Cancers of the lung and prostate rate among the top six cancers with white and nonwhite males, breast and cervical cancers are among the top six with white and nonwhite females, and melanoma represents one of the top six cancers with white females. Other types of cancer, including the various forms of leukaemia, although of lower incidence, are nonetheless causative for serious concern. The problem will be aggravated in the coming years as increasing urbanization is expected to create environmental and dietary conditions conducive to further initiation and spread of cancers; it will be further compounded by the finding that cancerous afflictions occur in approximately one third of all male and female adult breadwinners in the age bracket of 15–54 years. A novel factor now coming into play is the rapid spread of AIDS; immunodepressed patients will be at special risk to develop early cancerous lesions, and virus-related cancers may well find particularly suitable targets among such patients.

Chemotherapy constitutes an important cancer treatment modality, alone or in conjunction with other treatment regimens. However, despite much progress in drug research, results on average have been highly discouraging. Let us pause for a moment and examine this unsatisfactory state-of-affairs more closely.

Presently used anticancer drugs suffer from a multitude of severe pharmacological deficiencies, all of which continue to contribute to the limitations of effectiveness generally experienced in current clinical practice. Typical pharmacological shortcomings include the following: (i) *lack of cell specificity*, with ensuing drug distribution into both normal and transformed cells, that is, in consequence, drug application will be excessively wasteful; (ii) *inadequate water solubility*, hampering swift, and efficacious drug distribution in the body's aqueous fluid system, with resulting enhanced exposure to macrophage activity; (iii) *decreased serum half-life* as a consequence of catabolism, protein binding by the reticuloendothelial system, or efficacious excretion mechanisms; (iv) monophasic *salt-like* or *charged structure*, inhibiting membrane penetration and cell entry through normal passive diffusion, and resulting from these deficiencies, only a small fraction of the medicinal agent will successfully enter intracellular space for interaction with nuclear DNA or proteinaceous constituents; (v) *excessive systemic toxicity*, which grossly diminishes therapeutic drug efficacy; and (vi) lack of long-term efficaciousness because of inherent or induced *drug resistance*. Both (v) and (vi) are clearly the most aggravating factors added to abovementioned deficiencies, they generally necessitate premature discontinuation of drug-specific therapy, and thus, tend to reactivate tumorous or metastatic growth. As an overall consequence of the cited drug deficiencies, periods of regression tend to be limited, relapses occur frequently, and complete cures of most types of cancer by chemotherapy alone are still the exception rather than the rule.

A rather discomforting outlook indeed! Why then bring up this gloomy topic? Will you, the thoroughly depressed reader, not be prompted to disembark speedily from the wrong ferry boat and run for greener pastures elsewhere? The answer: worldwide efforts in biomedical research are striving to overcome existing chemotherapeutic hurdles through the expediency of developing efficacious cancer-fighting tools, and it is precisely this global development drive in which macromolecular compounds are crucial participants and find their true and foremost destiny. In Section 2.2 we will explore the background to this unique role played by polymers and their outstanding capabilities as delivery vehicles in the chemotherapeutic treatment of cancerous diseases.

### 2.2. The polymer-drug conjugation concept

**Figure 1:**
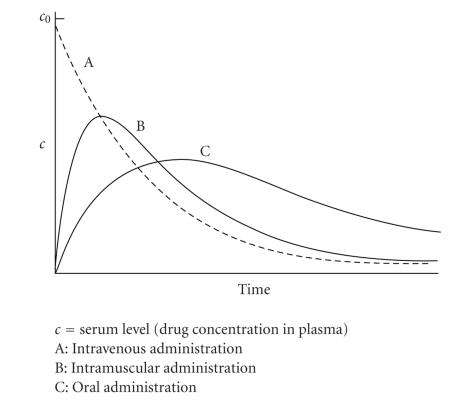
Drug serum concentration versus time for three administration routes.

To begin with, using text book knowledge, we will examine a typical medicinal agent's pharmacokinetic fate as it unfolds upon the drug's administration by the common intravenous, intramuscular, or oral techniques. For these, Figure 1 depicts exemplifying curves, given in terms of the agent's serum concentration as a function of postadministration time. In typical fashion, the intravenous route (curve A) delivers highest initial concentrations, which decrease more or less asymptotically with time, ultimately to reach zero-concentration level. Intramuscular administration (curve B) needs time for the curve to ascend from zero to a maximum, after which it gradually descends to zero. The oral route (curve C), while likewise providing a concentration maximum (generally below that of curve B), requires time extension for that trend, if one considers the long and obstacle-cluttered gastrointestinal pathway the drug has to follow before entering the blood pool.

**Figure 2:**
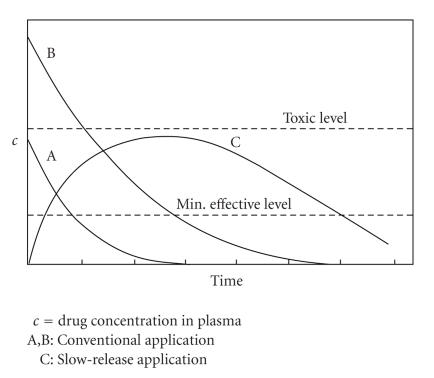
Drug serum concentration versus time for two different doses.

At this point, we learn that for any administered drug system there are two critical serum concentration limits, represented by the dashed lines in Figure 2: a lower limit below which the drug has ceased to produce any therapeutic effect, and an upper limit above which the drug exerts various forms of toxicity in the organism. For a drug employed via intravenous injection, which is the administration mode favored for polymer-drug conjugates, curve A allows for drug application devoid of toxic side effects, yet providing limited effectiveness time. In contrast, curve B, representing an increased initial dosis, yields a considerably extended effectiveness period; this, however, does happen at the cost of emerging toxicity. Hence, for a medicinal agent to remain therapeutically efficacious without toxic effects, its serum concentration must stay between the dotted lines over an optimally extended time span as schematically depicted by curve C.

**Figure 3:**
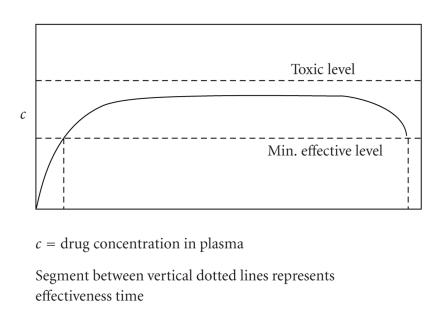
Drug serum concentration versus time for slow-release modality.

An obvious approach toward achieving this goal of continuous, nontoxic bioactivity, although being tedious and cumbersome, would involve repetitive drug administration within carefully identified time intervals and with doses adjusted so as to maintain serum concentrations just below toxic levels. Better still, one might design a drug delivery construct that would, upon a single bolus administration, allow for a slow and gradual intraserum release of the agent from some sort of depot system. An optimally efficacious concentration trend for this model is schematically shown in Figure 3.

Needless to say, the pharmaceutical community, far from being asleep, has over the past two decades focused research activities on a massive scale preferentially on this kind of drug delivery system, generally with highly promising outcomes. Thus, microencapsulated drugs in the form of microspheres, nanospheres, and liposomes have been developed and successfully tested, and so have numerous implantable matrices capable of liberating embedded drugs into the body's fluid system and other body compartments through bioleaching, bioerosion, or simply through matrix biodegradation. These amply discussed drug delivery systems [1–3], all aiming at enhanced therapeutic effectiveness of medicinal agents and by-and-large quite efficacious in their own right, fall outside the definition of the narrow term of polymerdrug conjugation and, hence, outside the scope of the present text.

Deviating in its *modus operandi* from the aforementioned drug delivery constructs, the drug conjugation modality embodies a symbiotic union between two equally contributing partners: the polymer acts as a carrier and transport vehicle (it “knows” how to overcome biological hurdles and deliver the drug load safely at the designated terminal), whereas its “partner in healing”, the drug proper, enjoys the safe ride and exerts its bioactivity in the target tissue. The notion of pairing up a bioactive agent with a transporter vehicle, born in the early 1970s, was forcefully advanced by Ringsdorf [4], who defined and refined this paradigm in palpable terms and set in motion broad-based development activities in numerous laboratories, on the strength of which the notion has by now matured to a highly sophisticated pharmaceutical tool. It utilizes the advantageous pharmacokinetic behavior of polymeric compounds in the mammalian body, paired with their enormous compositional versatility allowing for near-limitless structural modifications of these large molecules. In practical terms, the technique comprises the bioreversible binding (*conjugation*) of a bioactive agent, typically an antitumor drug, to a water-soluble macromolecular carrier molecule designed and synthesized in strict conformance with pharmaceutical guidelines. Specifically, the carrier polymer will be composed of the following: (i) subunits containing some molecular entity permitting facilitated cell entry; (ii) other subunits equipped with intra- or extrachain water-solubilizing groups; (iii) still other units featuring a homing device capable of directing the polymer-drug assembly, the *conjugate*, selectively to the target tissue; and (iv), most importantly, units equipped with functional groups suitable for the critical conjugation step involving bioreversible drug binding to the polymer. The conjugate represents a prodrug, from which the active agent is hydrolytically or enzymatically released into the predestined biological environment, which, for anticancer action, are the cytoplasmic lysosomes and cell nuclei. A conjugate conforming in structure to those specifications will demonstrate therapeutic superiority on account of the following factors.

- Even if insoluble in water, the medium providing the circulating blood pool and other fluids, the intraveneously (IV) or intraperitoneally (IP) administered polymer-anchored drug, now perfectly water-soluble, will be swiftly carried into central circulation or the intraperitoneum. This will ensure efficient distribution of the bound drug in the fluid system, a requirement most vital for drug transport within the body.
- While in transit in the central circulation system, the polymer-bound drug, notably if additionally modified by inclusion of oligo(ethylene oxide) units, will experience temporary protection from serum protein binding, enzymatic attack, and other scavenging processes. As a result, renal clearance (i.e., excretion through the kidneys' globular system) will be minimized, while serum life-time and drug bioavailability will be extended as part of a major change in biodistribution.
- The dreaded toxic side effects invariably caused by the common, nonpolymeric medicinal agents will be minimized through carrier attachment, as now the “anchored” agent will be constrained in body and organ distribution while in transit.
- The connecting groups incorporated into the polymer-drug linker for ultimate drug release can be uniquely designed so as to remain intact while the conjugate is on its way to target in the body's ever so slightly basic (ph∼7.5) environment, yet to undergo biofission once the carrier-drug assembly has reached intracellular (specifically lysosomal) space. Such biofission may be mediated by the lysosomal proteolytic enzymes, or else may simply follow a hydrolytic pathway, taking advantage of the acidic (ph∼5) intralysosomal environment. Reduced to monomeric size, the agent so liberated will now be able to undergo extravasation through penetration of the lysosomal membrane (a transport step not generally available to a macromolecular compound) and head for its ultimate destination, which for most anticancer drugs is the cell's nuclear DNA.
- The presence of a “homing” (i.e., actively targeting) group will lead to enhanced drug affinity for the transformed, that is, cancerous cell, thus facilitating guided drug transport. To exemplify this process, we wish to take a look at a cancer-targeting system featuring galactosamine as the homing device. This carbohydrate shows affinity for the asialoglycoprotein receptor in hepatocytes and thus, if attached to a cytotoxic agent, directs the latter efficiently to liver tissue, thereby providing cell-killing action against hepatomas, a form of liver malignancies. Other successfully used homing groups are represented by cationic moieties, tracking the conjugate preferentially to neoplastic tissue as a consequence of electrostatic attraction to the negative surface charge displayed by many cancer cells. Still other homing systems utilize the affinity of monoclonal antibodies for specific cancer-associated antigens. This research area, while still hampered by pharmacological problems of solid-state insolubility, immunogenicity, and in-transit vulnerability to side reactions and degradation, has proved eminently promising, and considerable progress has been achieved in recent years.
- The polymer-drug conjugate will experience facilitated endocytotic cell entry, thus circumventing potential problems caused by drug polarity or ionicity in the normal process of membrane crossing by passive diffusion common to nonpolymeric solutes. The mechanism is pinocytotic in nature and thus not subject to the limitations imposed by the reticuloendothelial system on phagocytotically captured particulates. Ideally, adsorptive pinocytosis is utilized by the conjugate for increased efficiency of translocation, and cationic polymers are good examples of compounds so translocated. In cases where excessive P-glycoprotein-mediated drug efflux from endocytic space and consequent resistance problems have developed, the pinocytotic cell entry mechanism, through replenishment of the intracellular drug pool, assumes unique importance as a means of counteracting the dreaded phenomenon of drug resistance and restoring chemotherapeutic drug activity.
- Polymer conjugates, in common with macromolecules in general, tend to accumulate in solid tumors because of enhanced intratumoral vascular permeability, allowing for substantial leakage of the polymeric molecules into the tumor tissue. Whereas, in normal tissues, macromolecules in interstitial space are efficiently recovered and eliminated by the lymphatic system, such lymphatic clearance is grossly retarded in tumorous tissue and so represents a weighty factor contributing to the tumoritropic characteristics of macromolecular compounds. This *enhanced permeability and retention* (EPR) effect associated with polymers provides passive targeting and renders polymer-drug conjugate administration more efficacious while reducing systemic toxicity in other organs.
- Through the expediency of introducing biofissionable segments into the backbone, a polymer can be rendered biodegradable, thus effecting its gradual removal from the body in the form of fragments qualifying for excretion through the body's normal waste removal systems. This process is crucial because high molecular-mass carriers are not readily eliminated from the body and in the longer term may cause additional toxicity.

**Scheme 1:**
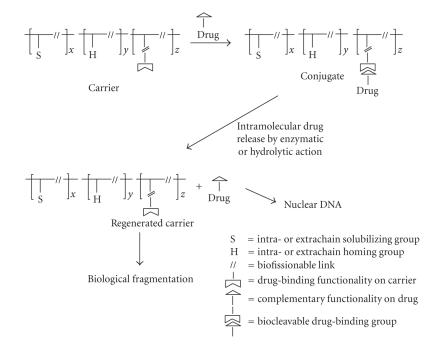
Pharmacokinetic *in vivo* pathway of polymer-drug conjugate.

A general flow chart depicting the pharmacokinetic pathway of a carrier-anchored antineoplastic agent constructed in conformance with the aforementioned specifications is schematically reproduced in Scheme 1. Most of the earlier papers in the field were concerned with natural macromolecules as carriers, such as carbohydrates or proteinaceous compounds; yet, as pointed out before, immunogenicity and backbone toxicity, premature biodegradation (alternatively, strong resistance to biodegradability), poor stability in the solid state, and other detrimental features are militating against major developments on that front. More recently, in line with the pharmaceutical guidelines discussed further above in this section, synthetic polymers have taken the lead in drug conjugation studies for reasons of synthetic versatility and reproducibility, improved control of physical and chemical behavior patterns, as well as minimization of systemic toxicity and immunogenic properties. Parenthetically (no metal-based constituents being involved here) we wish to introduce the reader to a related, rapidly growing branch of drug conjugation science known as *gene therapy*. In this therapeutic modality, synthetic polymers are used in place of viral vectors as vehicles for the *in vivo* transport of specific nucleic acid agents for the therapeutic manipulation of pivotal genes involved in malignancies and other diseases. Despite existing pharmacological shortcomings, gene therapy is bound to take its well-deserved place in forth-coming therapeutic technology developments [5, 6]. The immensely wide-ranging drug conjugation topic has been amply surveyed over the years in the literature, and some of the most outstanding reviews both of yesteryear [7, 8] and up-to-date [9–14], are recommended to the reader for in-depth studying.

Having thoroughly digested the fundamental principles of polymer-drug conjugation and the significant pharmacological benefits to be derived from this mode of drug delivery, we are ready now for a lecture on the practicalities. Out of a large number of variously structured carrier types developed over the past decade, Section 3 will introduce just three types that have proved to be the predominant role players in the drug conjugation domain, and their structural peculiarities will be highlighted as a helpful prerequisite for the discussion of the drug anchoring strategies covered in Section 4.

## 3. THE LEADING CARRIER SYSTEMS

The design and construction of a drug carrier represents a pivotal task in polymer-drug conjugation as both the anchoring chemistry and the therapeutic activity of the derived conjugate depend critically on the carrier's solubility behavior and resulting “staying power” in solution upon drug conjugation. Steric accessibility and reactivity of the anchoring sites additionally contribute to the carrier's functional efficacy. A nontoxic chain construction amenable to slow biodegradation in the spent state (i.e., upon drug liberation) is a further requirement for optimal efficaciousness. Lastly, high main-chain flexibility depending on freely rotating single bonds in the backbone and the absence of aromatic or heteroaromatic, sterically blocking units, will be required as this will increase the entropy of solution, thus reinforcing the aforementioned staying power of the assembly in solution. In the following text we will find out if, and to what extent, these prerequisites are met in the structural compositions of the three selected carrier types: (1) the *α*,*β*-DL-polyaspartamides, (2) the poly(amidoamines), and (3) the 2-hydroxypropyl-methacrylate polymers (HPMA). In addition, polyamides obtained by polycondensation processes will cursorily be discussed because of their sporadic use as carriers.

### 3.1. *α*,*β*-DL-polyaspartamides

**Scheme 2:**
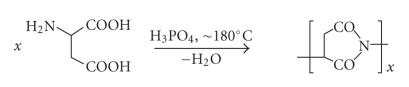
Synthesis of polysuccinimide from aspartic acid.

This carrier type has moved into the limelight of polymer research some three decades ago [15–17]. It is readily accessible synthetically by high-temperature condensation polymerization of DL-aspartic acid in orthophosphoric acid medium, leading to a polysuccinimide intermediate (see Scheme 2).

**Scheme 3:**
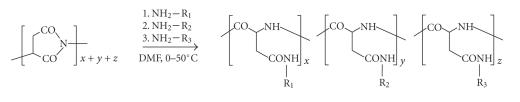
Aminolytic ring opening in polysuccinimide.

In subsequent polymer-homologous reactions conveniently performed in a dipolar aprotic solvent such as N,N-dimethylformamide, the intermediary polysuccinimide is treated sequentially with two amine nucleophiles (NH_2_–R_1_, NH_2_–R_2_; occasionally a third amine, NH_2_–R_3_, may be employed in this operation), whereby imide ring opening gives rise to the generation of the ultimate polyaspartamides [18, 19]. These linear polyamides are composed of randomly distributed aspartamide repeat units bearing N-attached substituents (R_1_, R_2_, etc.) as introduced by the amine nucleophiles chosen. Scheme 3 depicts this sequence of ring opening steps.

It should be noted that here and in subsequent polyaspartamide representations, only the *α*-peptidic forms are shown for convenience, although the isomeric *β*-forms, with two carbon atoms (rather than one only) separating –CO and NH–, are also present.

**Figure 4:**
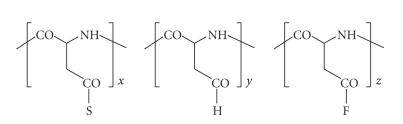
Copolyaspartamide equipped with solubilizing, target-directing, and drug-binding moieties.

Let us now redraw the structural arrangement in Scheme 3 by substituting a solubilizing entity S for NH–R_1_, a homing device H for NH–R_2_, and a drug-binding functional group F for NH–R_3_ (see Figure 4).

It should be evident from the detailed discussion in the foregoing of structure-function relationships that polyaspartamides complying with this schematic representation will be able to provide an excellent “workhorse” service in drug conjugation studies. Specifically, the following hold. (i) The vital prerequisite of water solubility will be afforded by the numerous freely rotating aliphatic main-chain bonds and, in particular, by the extrachain S units chosen so as to contain *tert*-amine, hydroxyl, or, perhaps, oligo(ethylene oxide) groups [20]. These functionalities will be prone to aquation, that is, hydrogen bond formation with the aqueous solvent, and/or protonation with generation of cationic sites. (ii) Steric accessibility of the anchoring site F is optimized by insertion of a suitably long (say, 8–10 atomic constituents) aliphatic spacer link between F and backbone, thus reducing any steric hindrance potentially provided by the main chain proper. (iii) The chain construction has been rendered nontoxic through use of aspartic acid as the principal molecular backbone source. (iv) The chain is biodegradable hydrolytically at the CO–NH links for catabolic fragment elimination through the globular system of the kidneys upon drug release, a vital detoxification process in light of the fact that, as mentioned before, large nondegradable macromolecules, such as vinyl-type polymers and other carbochain macromolecules, strongly resist excretion and tend to induce toxicity. Such backbone degradation must be slow and controlled, however, so as to ensure stability during serum exposure, yet fragmentation in the endosomal compartment. (See also remarks in Section 2.2). In the polyaspartamide structure, these conditions are ideally met: in the serum environment (pH∼7.5) only minimal hydrolytic CO–NH bond fission is observed, whereas in the endosomal space (pH∼5) such fragmentation proceeds as expected, albeit slowly. Superimposed on hydrolytic bond fission is the process of enzymatic bond braking, effective both at main-chain NHCO sites and, to a variable extent, also at drug-binding links. Whereas natural polypeptides are prone to rapid degradation (*unzipping*) as a consequence of *α*-peptidase activity in the serum, such unzipping is grossly impeded by the presence of D-configurated CH groups and *β*-peptide units in the chain that are inert to *α*-peptidase attack [21]. Section 4 will show how proper use can be made of these structural features.

### 3.2. Polyamidoamines

**Scheme 4:**
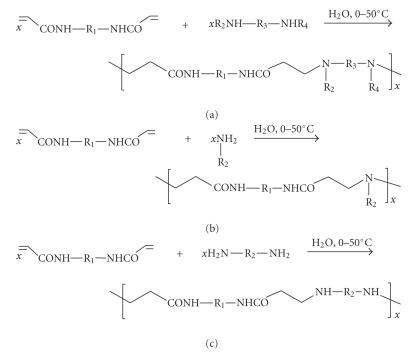
Different modes of Michael addition polymerization of a bisacrylamide.

Pioneered in Ferruti's laboratory [22, 23], these macromolecules have proved as versatile as the polyaspartamides, allowing for the construction of a multitude of unique carrier compositions. In the simplest mode of synthesis, a monomer equipped with two activated vinyl groups as in a bis(acryloylamido) compound exemplified by methylenebisacrylamide or bisacryloylpiperazine, is allowed to undergo aqueous-phase Michael addition polymerization with a comonomer that either contains two secondary amino groups for single substitution on each N (see Scheme 4(a)) or else features a primary amine (see Scheme 4(a)), the latter now being doubly N-substituted in the process. The reactions are preferably conducted in aqueous solution and give rise to polymer structures comprising both amide and amine functionalities. In the author's laboratory these studies were extended to include comonomers featuring two primary amino groups that are allowed to enter polymerization by single-step Michael addition at each –NH_2_ under modified, carefully controlled conditions. This will produce polyamidoamines possessing secondary amino groups in the main chain (see Scheme 4(c)), which, in turn, are destined for drug binding [24]. With R_2_ standing for an ethylene residue, the resulting polymer possesses an intrachain –NH–(CH_2_)_2_–NH segment important for *cis*-N,N chelation of platinum, as will be shown in Section 4.

**Scheme 5:**
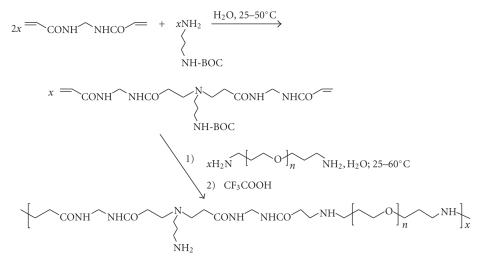
Michael addition polymerization involving mono-N-protected diamine.

A special case of sequence (b) in Scheme 4, in which a mono-N-protected diamine takes the place of H_2_N–F_3_, leads to a polymer possessing the mono-N-protected moiety as a side group. This strategy thus compels primary diamine monomers to be incorporated through just one amine functionality instead of two as in (c). Subsequent N-deprotection then provides a polymer featuring a free –NH_2_ side-chain terminal directly available for drug anchoring [25]. Scheme 5 depicts this reaction course (*n* = 3–21).

It is obvious from these described synthetic strategies that the resultant polyamidoamines in all cases are linear and follow the established rules of construction, in so far as one may vary the intrachain-type segments as well as—more usefully—the extrachain-type side groups, in an effort to provide the vital functions of solubility, target homing, and drug conjugation. In Section 4 we will learn more about the versatile use of the amidoamine polymers.

### 3.3. N-(2-hydroxypropyl)methacrylamides (HPMA)

The HPMA polymers have followed a glorious development path since early beginnings in 1970s [26]. On the strength of preceding methacrylate polymer research in numerous laboratories, this polymer class, developed almost singularly in the collaborating laboratories of Kopeček (synthesis) and Duncan (biomedical evaluation), has by now most successfully entered the forum of global research in polymer therapeutics [27–29].

**Scheme 6:**
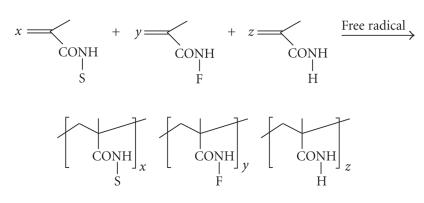
Synthesis of methacrylamide polymers.

The synthetic approach, illustrated in Scheme 6, involves the free-radical copolymerization of two or more methacrylamide monomers bearing substituents S for provision of water solubility (almost always a 2-hydroxypropyl moiety), other substituents, F, for drug anchoring reactions, and still other functionalities, H, as required for specific purposes serving facilitated cell entry, homing to the cancerous target tissue, or in vivo detection and tracing.

**Scheme 7:**
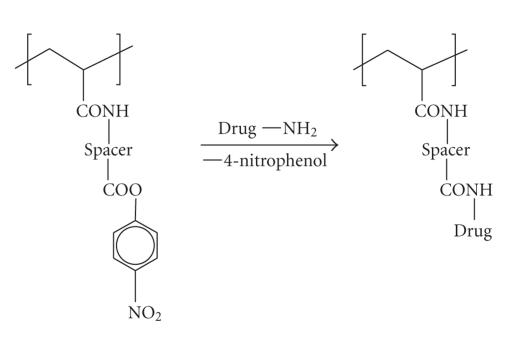
Drug conjugation with HPMA copolymer.

The polymers are thus characterized by a carbochain-type backbone composed of substituted 1,2-propylene repeat units, and these are randomly distributed along the main chain. In contrast to the polyaspartamide situation, all basic functionalities are generally preintroduced into the monomers and are automatically polymer-incorporated as the free-radical propagation proceeds. In order to facilitate the drug conjugation step, the respective precursor monomers are methacrylamides substituted with a spacer-separated *para*-nitrophenolate group, the latter acting as a convenient leaving group in the ultimate addition-elimination step leading to the introduction of amine-functionalized drug species with loss of *para*-nitrophenol. A nitrophenolate-terminated repeat unit and its reaction product, the drug-conjugate unit, are shown in Scheme 7.

Selected HPMA conjugates derived from the here-described carriers will be dealt with in Section 4.

### 3.4. Polyamides prepared by polycondensation reactions

**Scheme 8:**
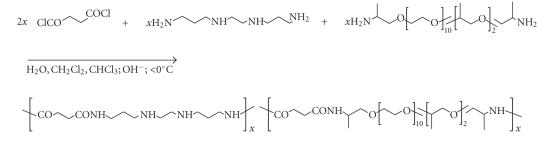
Synthesis of polyamides by interfacial polymerization.

Although of less importance, polycondensation products resulting from interfacial or high-temperature condensation reactions should be included here because monomer variation results in a variety of carrier structures featuring introduction of –NH− groups useful for drug conjugation. Typically, exemplified in Scheme 8, a diacid chloride, such as succinyl chloride, is allowed to react interfacially with a diamine like 1,2-bis(3-aminopropylamino)ethane or the solubilizing commercial Jeff ED-600 (an oligo(ethylene oxide) terminated at both ends with a primary amino group) [30]. High-temperature solution polymerization in polyphosphoric acid provides analogous polyamides, if free diacids are employed in place of the acid chlorides [31]. The –NH–(CH_2_)_2_–NH– segments in these structures have conveniently been introduced for platinum chelation as pointed out before.

## 4. SELECTED CARRIER-BOUND DRUG MODELS

Having acquired by now the requisite conceptual tools, we are duly prepared for the ultimate topic: the strategies required to put the carrier-drug assembly together along biomedically prescribed routes. As we delineate the scope of this effort in the forth-coming discussions, we are guided by the requirement to confine the treatment to metal-based polymer systems and, there again, to those systems with a medicinal, specifically therapeutic application potential. Among the considerable number of metal-containing macromolecules that have attracted the bioscientists' interest, we will uniquely focus our attention to the two most actively researched classes: the metal-coordination polymer class belonging to the platinum drug family, and the organometallic class of ferrocene-type iron-containing conjugates. Both classes, as we will see, turn out to be just the right types of “guinea pig” as we set out to explore the broad field of medicinal agents conjugated to macromolecular adjuvants. In addition (you guessed it!), it so happens that it is the development of these two classes to which the author's group has been particularly devoted over the past four decades. Other metals, such as titanium or ruthenium, continue to play a role in the field marginal enough as yet to permit us to ignore them within the scope of this text.

### 4.1. Polymer-platinum conjugates

**Figure 5:**
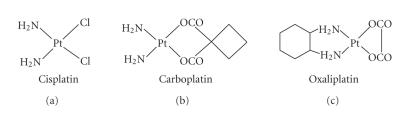
Leading platinum drugs in clinical use.

As metal-based medicinal agents found their way into the oncologist's arsenal of antitumor drugs, several decades ago, platinum containing compounds immediately moved to the forefront with the advent of the prototype, *cisplatin* (*cis*-diamminedichloroplatinum(II)), and its second-generation cousins, *carboplatin* and *oxaliplatin* (see Figure 5).

Their general mechanism of action involves the formation of aquated species upon parenteral administration and subsequent intra- and interstrand crosslinking with intracellular DNA, leading to irreversible lesions in the double-helix and ultimate cell death. The compounds are highly potent against numerous malignancies *per se* and in combination with other antitumor drugs. Set against these highly acclaimed performance criteria, the platinum compounds, just like the metal-free drugs, suffer from severe pharmacological shortcomings, curtailing clinical effectiveness. Thus, residence times in central circulation are generally short, and this is paired with rapid excretion through the urinary tracts and consequent unpredictable variation of drug concentration. Drug action is nonselective, normal and transformed cells being equally affected; this leads to significant, dose-limiting acute and chronic toxicities, and in consequence, therapy discontinuation will frequently be required long before regression-free cure rates are attained. Lastly, and most importantly, various forms of drug resistance tend to build up in the cancerous tissue, which, again, requires treatment termination. In view of these serious deficiencies, active research worldwide has been focused on structural modifications designed to enhance the overall performance profile [32–35]. The polymer-drug conjugation modality is playing an outstanding part in these research endeavors, and, as we will see in the following section, encouraging progress has been made until now. Before going into details, let us remember that the present treatise, far from presenting a comprehensive review, is intended, first and foremost, to provide a didactic lecture on the topic; hence, it will cover only a small number of exemplifying conjugate types selected to mirror general trends in this rapidly expanding field of pharmaceutics. For the student wishing to “dig deeper”, Carraher's excellent review chapter with original literature quotes is strongly recommended [36].

#### 4.1.1. Metal coordination involving carrier-bound nitrogen donor ligands

**Scheme 9:**
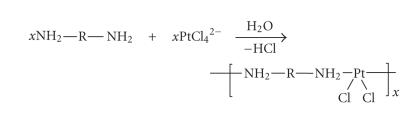
Polycondensation of diamines with the tetrachloroplatinate dianion.

By strict definition of the polymer-drug conjugation concept, the presynthesis of a suitable carrier polymer of biomedically “approved” structure is followed in another reaction step by the bioreversible attachment of the drug. Before we study the exemplifying macromolecules compliant with this definition of drug conjugation, it is only fair to Charles Carraher, the pioneering scientist, that we are acquainted first with a separate synthetic approach deviating from that routine, yet significant in its own right because it is one of the earliest major assaults on the problem of providing biomedically important metal compounds in polymeric form. This approach, developed in meticulous detail in Carraher's laboratory, embodies the aqueous-phase polycondensation of the tetrachloroplatinate(II) dianion with diamines, giving polymers with the metal coordinated directly into the main chain in *cis*-diamine fashion, as shown in Scheme 9. The link R in this process stands for a variety of aliphatic, aromatic, and heteroaromatic units, or simply for a direct bond, and molecular masses attain the 10^6^ Dalton level. Biological test data, notably for antiviral and antiproliferative performance against a number of cancer cells, at that time demonstrated a “leap forward” in platinum-drug cancer therapy.

**Scheme 10:**
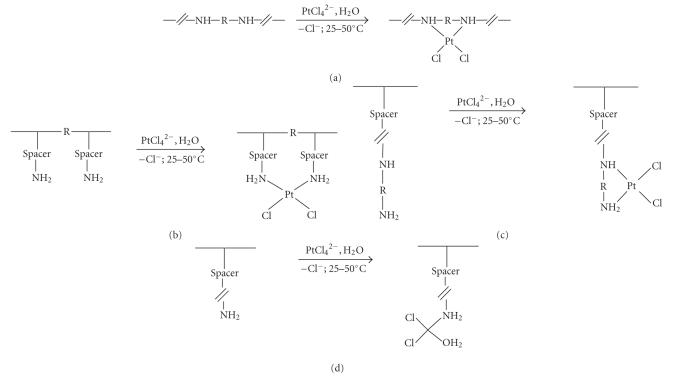
Platination of amine-functionalized carriers.

This brings us to the main stream of drug conjugate development: the polymerhomologous drug anchoring to presynthesized carrier polymers. In this principal mode, polymer attachment of the square-planar structural element of *cisplatin* can be achieved *via* several reaction paths, in the design of which care must be taken to include suitable cleavage sites in the assembly for *in vivo* liberation of the bioactive agent from the carrier vehicle. In principle, two approaches of metal-binding are available. In the first, the metal center is bound *via* amine ligands attached to the carrier. In the second mode, the metal is polymer-bound *via* the leaving group ligands. In the first case, the result will be a structure comprising a Pt center tightly bound to the carrier, and release of the active complex will require cleavage of a suitable anchoring segment in the side chain. This is a time-dependent process, and the conjugates so constructed should prove most efficacious in long-term administration. In the second case, the active complex is released from the conjugate through (generally hydrolytic) cleavage of the bonds connecting the metal with the leaving groups, resulting in aquation. Release rates will be higher in this model, which should, hence, find optimal utilization in short-term administration, whenever a boost is required for rapid effects. Let us look at the first case: conjugation *via* carrier-bound amine ligands (see Scheme 10).

**Scheme 11:**
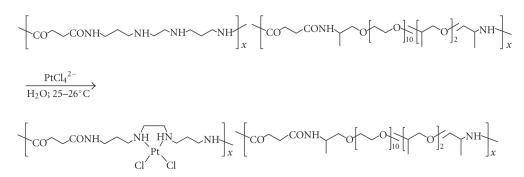
Platinum atom coordinated by intrachain ethylenediamine segment.

In the simplest fashion (type A, with R = CH_2_CH_2_−), the >PtC*ℓ*_2_ moiety is incorporated into a carrier structure containing ethylenediamine as a main-chain segment. Such carriers may be prepared by Michael addition polymerization with selected tetramine comonomers, and Scheme 11 provides an illustrating example. A related metal bonding pattern exists in certain polyethyleneimines prepared by Carraher's group. In conjugates of these structures the *cis*-diamine ligand system is strain-free and thus smoothly generated.

Platination of carriers of type B (see Scheme 10) leads to conjugates in which the entire *cisplatin* skeleton is located outside the main chain. With R standing for a direct bond or, less favorably, no more than a single atomic constituent, metal coordination through chelation with both amino groups would appear to be sterically feasible. Exemplifying product structures have indeed been obtained in Carraher's laboratory [36] by platination of a vinylamine/vinylsulfonate copolymer and of the natural (and hence, biodegradable) polymer chitosan. A special case of type B has been reported by Howell and Walles [37]. These authors *inter alia* presynthesized a *cisplatin* analog with both NH_3_ ligands replaced by the two NH_2_ groups in 1,2-diamino-4-hydroxybenzene. This reactant was then allowed to bind to poly(vinylpyrrolidone) by molecular complexation, that is, in a form permitting its release from the carrier by simple dissociation [37].

**Scheme 12:**
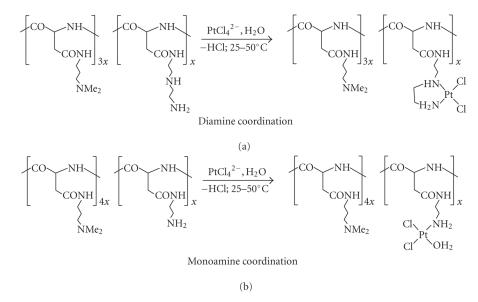
Platinum conjugation through carrier-attached amine donors.

Appreciably more research has been performed with conjugates falling into the pattern of types C and D (see Scheme 10). In the author's laboratory, polyaspartamides prepared per Scheme 3 (yet including only two R-substituted units) were converted to platinated species by treatment with tetrachloroplatinate dianion in aqueous phase. The two platination runs in Scheme 12 exemplify this reaction type. Whereas the upper reaction leads to a conjugate with *cis*-N, N′-coordinated platinum [38, 39], the lower one gives rise to the formation of a structure wherein the metal is polymer-bound through a single N donor ligand [40]. In both cases, the first depicted, potentially cationic repeat unit provides both solubilizing and homing functions. Cell culture tests showed both conjugate types to be equally antitumor-active, and while with the upper conjugate ranked first in a series of cytotoxicity tests [41], the lower one ranked first in an *in vivo* toxicological study, demonstrating some 100-fold lower toxicity than determined for the *cisplatin* standard [42].

Similar platination patterns were reported from Ruth Duncan's prolific laboratory (reviewed [36]), with HPMA polymers used as the carriers. Broad-based biological investigations provided excellent results with respect to release rates, toxicological behavior, and cell-killing properties both *in vitro* and *in vivo*.

**Figure 6:**
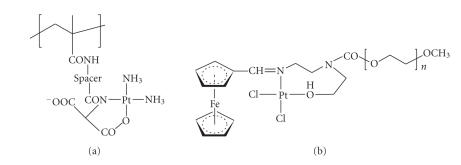
Platinum conjugation through repeat units featuring carrier-attached N- and O-donors.

Somewhat related to type D is a conjugate synthesized by Gianasi et al. [43] and further investigated by Howell [44, 45], in which only one nitrogen donor ligand, in cooperation with an oxygen donor, partakes in platinum coordination (See Figure 6(a)). The compound, an HPMA derivative, is noteworthy as it features and N,N,N′,O-coordination square around the Pt atom. The compound underwent *in vitro* and Phase I clinical tests with highly promising results, low toxicities being paired with excellent antiproliferative activities [44, 45].

A differently structured polymer type featuring N,O-coordination of the metal has just been reported by Bariyanga [46]. Representative compounds contain the Fc−CH=N(R)−Pt(Cl^2^)−O− segment (Fc = ferrocenyl) connected to a poly(ethylene oxide) terminal (see Figure 6). These polymers are thus characterized by the presence of both a ferrocene and a platinum complex. While no biomedical test data are available, it would be interesting to find out in future studies whether such compounds display cooperative cytotoxicity between the two drug species.

#### 4.1.2. Metal coordination through carrier-bound oxygen donor ligands

**Scheme 13:**
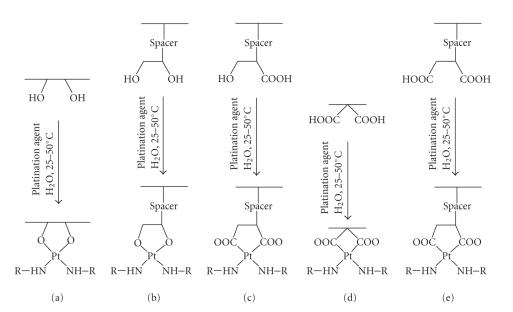
Platinum chelation patterns.

In the polymer described in this section the Pt complex is conjugated through two oxygen donor ligands provided by the carrier. Contrasting with those covered in the preceding section, these conjugates follow a release mechanism involving hydrolytic Pt bond cleavage, thus requiring no special biofissionable links in the carrier for drug liberation. In structures of this kind, the anchoring mechanism must be designed with a view to counteracting the inherently limited stability of the O−Pt bond. The preferred approach to this goal involves the construction of 5–7-membered chelate rings connecting the metal atom with the carrier-attached donor ligands. Scheme 13 offers acceptable choices, for which the literature provides exemplifying experiments (reviewed by Carraher [36]); whereas types A and D exemplify carrier binding through directly backbone-attached hydroxylato- or carboxylato-type oxygen donors, the types B, C, and E represent structures with spacers interposed between carrier backbone and oxygen donor. Further work will determine the extent to which biological performance will be affected by the coordinating atoms' special remoteness from the main chain.

**Figure 7:**
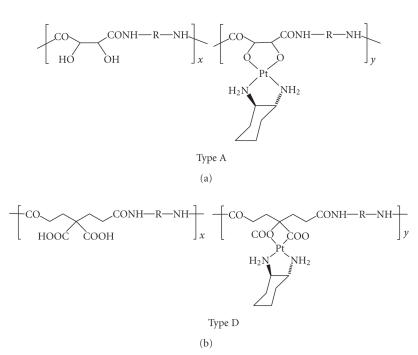
Examples of O,O-coordinated platinum in close main-chain proximity.

Figure 7 provides an example each of type A- and type D-derived conjugates without further comments on the synthetic methodology [47].

**Scheme 14:**
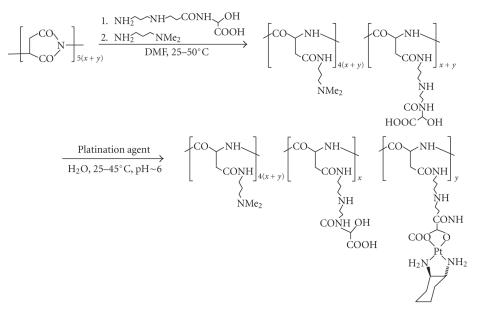
Synthesis of conjugate featuring extrachain-type 1,2-carboxylatohydroxylato coordination of platinum.

Considering now the B- and C-type platination reactions of Scheme 13, the literature provides numerous reaction sequences originating from uniquely functionalized carriers. Scheme 14 represents a prototype reaction starting out from polysuccinimide [47]. In all these exemplifying cases, the platination agent has been *trans*-1,2-diaminocyclohexanediaquaplatinum(II) dinitrate (DACH-Pt), a reactant preferred by us and others over the tetrachloroplatinate dianion on grounds of favorable biological behavior, notably the lack of crossresistance with *cisplatin*.

**Scheme 15:**
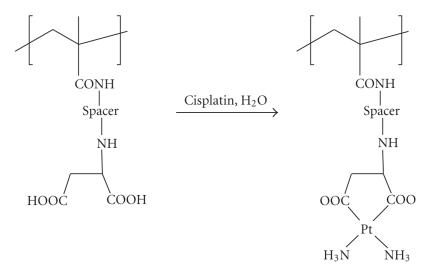
HPMA-derived dicarboxylato-coordinated platinum.

We will now focus our attention to the bonding pattern of type E (see Scheme 13) which underlies the largest body of recent carrier-Pt conjugation research. Thus, Ohya et al. [48] prepared a water-soluble poly(ethylene oxide) carrying a methoxy terminal and, at the other end, a propylmalonate moiety suitable for platination with hydrolyzed *cisplatin*. The release behavior was examined and the cytoxicity against lymphocytic leukemia cells was determined. Good activity, although slightly below that of *cisplatin*, was observed. A study by the afore-mentioned group of Duncan et al. produced numerous dicarboxylatoplatinum compounds, among others, HPMA-derived conjugates possessing carrier-attached dicarboxylato ligands [43]. Upon treatment with hydrated *cisplatin*, these converted to highly bioactive *cis*-dicarboxylato-chelated platinum polymers as exemplified in Scheme 15 for the affected repeat unit.

**Figure 8:**
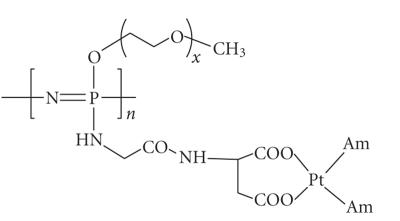
Simplified structure of polyphosphazene repeat unit bearing sidechain-coordinated platinum.

A recent patent, outstanding among the many studies of polymeric phosphazene drugs [49], describes polyphosphazene derivatives of dicarboxylato-coordinated platinum with activity *inter alia* against the gastric tumor YCC-3 [50]. A simplified repeat unit structure is reproduced in Figure 8.

**Scheme 16:**
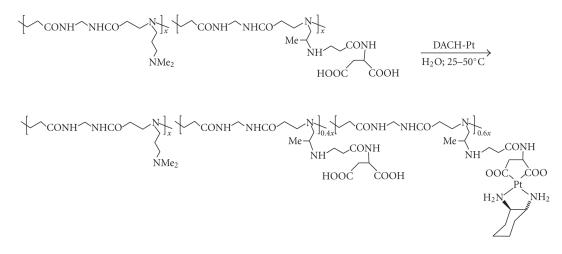
Polyamidoamine-derived dicarboxylato-coordinated platinum.

Numerous conjugates of type E have in recent years been synthesized in the author's laboratory, of which only a single example is reproduced in Scheme 16. The carrier educt in this reaction is a polyamidoamine equipped with a solubilizing N,Ndimethylaminopropyl side chain and an aspartic acid-derived dicarboxylate, and platination once more involves treatment with DACH-Pt [47, 51]. This polymer and related structures have shown outstanding antiproliferative performance, on the strength of which, a major development program has been initiated.

This section would be incomplete without a brief treatment of the comprehensive research efforts by the group of Bilha Schechter and collaborators. These workers at the Weizmann Institute of Science have been involved for some decades now in studies aiming at the provision of platinum-containing polymers antibody-modified for immunotargeting to human tumors. Representative compounds are a polyglutamic acid providing chelating capacity of type E (see Scheme 13) and some carboxymethyl derivatives of dextran with similar, albeit less structurally defined metal coordination patterns. Excellent biomedical test results on a broad front attest to the soundness of the development program in Schechter's highly productive laboratory. For in-depth reviews of the Weizmann group's activities, see Siegmann-Louda and Carraher [36], and Chen et al. [52].

### 4.2. Polymer-ferrocene conjugates

Contrasting with the platinum drugs, which have firmly established their prominent position as antitumor agents in clinical use, research in the realm of ferrocene-based chemotherapy is still at an experimental stage. However, biomedical test data, preliminary as they are, have shown overwhelming promise, stimulating comprehensive investigations currently in progress worldwide. The core organoiron compound, di-(*η* ^5^-cyclopentadienyl)iron(II), has been under scientific and technical scrutiny for more than half a century, and numerous topical surveys, including fundamental physical and chemical applications, have appeared in the literature (surveyed in two books [53, 54]). The fascinating ferrocene chemistry, pivotally involving the compound's redox equilibrium with its one-electron oxidation product, the ferricenium cation, has prompted wide-ranging investigations of this electrochemical ferrocene/ferricenium couple in the biological environment, with ultimate ramification into the topic of carrier-bound ferrocene conjugates.

The bulk of contributions to these research developments has materialized in the author's laboratory, as attested by more than 50 pertinent papers published by our group until now. The reader is referred to some reviews [54–57] covering this prominent development trend.

Let us look now in detail at the large family of polymer-bound ferrocenes designed for biomedical applications. As we embark on this theme, we realize that, in the “old days” of ferrocene polymer development spanning the 1950–1970 decades, macromolecular ferrocenes and other metallocenes had been synthesized in huge numbers (reviewed in the book [54] and three book chapters [58–60]), yet most of those were hydrophobic and lacked hydrosolubility and, hence, were not considered in later work. An entirely new concept had to be developed for the purpose of rendering any metallocene polymers qualified for biological examinations. That concept, as we learned in Section 2, originated a decade later. It embodies the technique of conjugation so successfully utilized in the design of polymeric platinum and other drugs, and in the present section it will be seen to be instrumental in the synthesis of carrier-bound ferrocene compounds.

Earlier exploratory studies [61] demonstrated the feasibility of carrier binding and provided a range of variously structured conjugates meeting the crucial requirement of water solubility. Ferrocenylamines, initial candidate agents for ferrocenylation because of their strongly nucleophilic functionality, were soon omitted on electrochemical grounds. Ferrocenyl-carboxylic acids, on the other hand, notably 4-ferrocenylbutanoic acid, emerged in those earlier investigations as suitably functioning compounds providing electrochemically stable ferricenium counterparts [62–64]. The butanoic acid derivative soon became the ferrocenylation agent of choice in all subsequent conjugation developments in preference over other chemically feasible coupling mechanisms. Again, the polyaspartamides introduced in Section 3.1 turned out to be the most versatile and functional carriers; equipped with NH_2_-terminated side chains, they soon became, and still are, the “workhorse” carrier polymers in our laboratory, conveniently undergoing coupling reactions in aprotic medium with the ferrocenyl acid via carboxyl group activation, or directly with the aid of strong coupling agents, such as HBTU (O-[1H-benzotriazol-1-yl]-1, 1,3,3-tetramethyluronium hexafluorophosphate). Scheme 18 shows a typical reaction step of this type, singled out here on account of the conjugate's particularly rewarding cytotoxic behavior; *in vitro* screens against the HeLa human cervix epithelial cancer revealed a cell-killing activity (on metal-to-metal equivalent basis) twice that of the *cisplatin* standard, and a fourfold superiority over *cisplatin* against Colo 320 DM, a notoriously drug-resistant human colon cancer line [65]. Interestingly, similar conjugates possessing nonbasic hydroxyalkyl side groups in place of the strongly basic *tert*-amine-terminated solubilizing and homing moiety of the drawn structure gave rather poor results, reflecting the lowered affinity to the cancerous cell (see Section 2.2) because of lacking basicity.

Among the great variety of other carrier types, we cite the primary amine-functionalized amidoamine polymer synthesized by Michael polyaddition (see Section 3.2); readily ferrocenylated with HBTU mediation [66], it converts to a conjugate featuring a long (8 atomic constituents) spacer connecting the metallocene to the main chain as depicted in Scheme 19.

On the other hand, a related amidoamine-type carrier characterized by an additional intrachain −NH−NH− group yet lacking the NH_2_-spacer side chain converts to a conjugate by direct N-acylation of the main chain [67] (see Scheme 20). Subjected to the same cell culture tests as cited above, this ferrocene-loaded polymer, again, performed distinctly better than the *cisplatin* standard.

Lastly, we mention an anchoring step involving O-acylation. To this end, various hydroxyalkyl-substituted polyaspartamides were treated in aprotic medium with ferrocenylbutanoic acid in the presence of N,N′-dicyclohexylcarbodiimide coupling agent and 4-(dimethylamino)pyridine (DMAP) catalyst [68]. Scheme 21 shows a representative run, with * x/y* varying from about 3 to 10 as a function of molar reactant ratios.

A conjugate of this type, with * x/y* = 2.6, subjected to an *in vivo* toxicity test, gave a maximum tolerated dose of 0.43 mmol Fe/kg, more than 20-times higher than determined for the *cisplatin* standard [69]. The polymer is thus some 20-fold less toxic than the standard, and comparable toxicity behavior is also displayed by selected amide-linked analogs. These findings, in conjunction with cell-killing properties, augur exceptionally well for the class of ferrocene conjugates as cancer-chemotherapeutic agents, and their future looks highly promising indeed.

## 5. SUMMARY AND CONCLUSIONS

Mankind, along its arduous development path from early hominids via *Homo erectus* to the present “status” of *Homo sapiens*, has never been spared the experience of bodily injuries and the suffering from debilitating or even lethal diseases. Yet the human spirit has at all times searched for therapeutic weapons to overcome, or at least deter, those inimical adversaries. Even at the sophisticated level of human enterprise attained by now, we have come to accept the limited efficacy of those therapeutic weapons against many serious infirmities and the pressing need for continued forceful research activities to eliminate existing threats to our well-being. Cancerous diseases stand out amid those threats as particularly dreaded adversaries, and on these we have focused our attention in the present discourse.

**Scheme 17:**
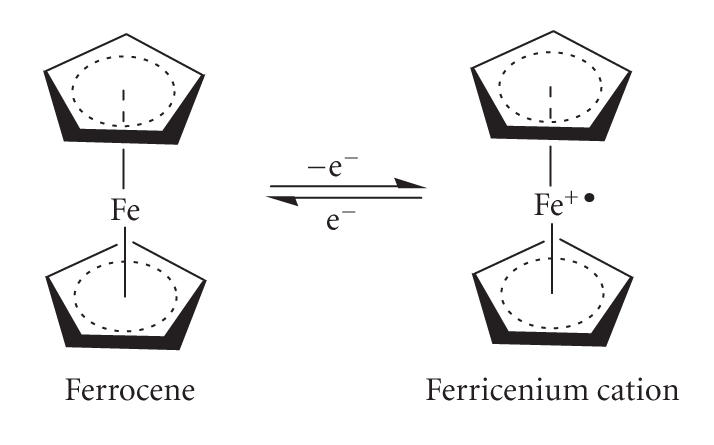
The ferrocene/ferricenium redox couple.

**Scheme 18:**
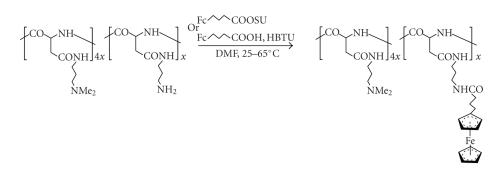
Polyaspartamide-derived ferrocene conjugate.

**Scheme 19:**
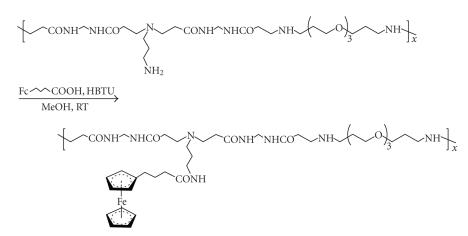
Poly(amidoamine)-derived ferrocene conjugate.

**Scheme 20:**
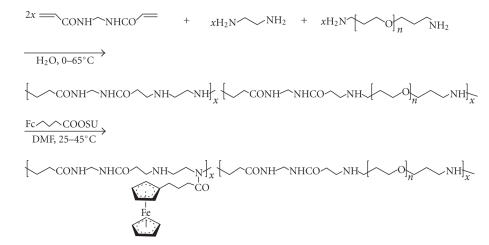
Ferrocenylation of amidoamine polymer featuring intrachain-type −NH− group.

**Scheme 21:**
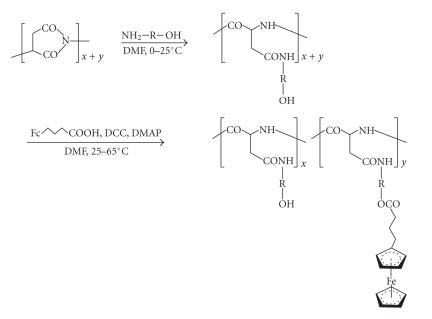
Ferrocenylation of polyaspartamide featuring extrachain-type hydroxyl groups; R = aliphatic segment.

We have acquainted ourselves with the cancer problem *per se*, with the current chemotherapeutic treatment limitations, and, getting into the core topic, with the promising and highly challenging present research endeavors in the field of polymer-drug conjugation. In doing so, we have followed the development of one of the leading polymer-based chemotherapeutic tools in the fight against cancer, with emphasis on platinum- and iron-based drug systems. We have found out that the expediency of using goal-designed, water-soluble, and biologically acceptable macromolecules as temporary transport vehicles for powerful, yet potentially toxic, and resistance-promoting medicinal agents has, beyond early doubts and objections, matured to a practicable technology. It is to be hoped that the facts and arguments here-presented will encourage many students of the biochemical and pharmaceutical sciences to participate in the global efforts to promote and refine the drug conjugation modality for the future benefit of the victims of neoplastic and other diseases Scheme 17.
